# Progress in Medicine

**Published:** 1897-11-20

**Authors:** 


### Progress in Medicine.

### OBSTETRICS.

(*Continued from page* 120.)

**Dysmenorrhœa.**—Opinions on this subject seem to differ as much as ever. Drummond Robinson[37] draws attention to the engorgement of the pelvic organs which occurs during the last part of the inter-menstrual epoch, and shows that this does not rest on a theoretical basis, but has been confirmed by actual observation. He considers that uterine contractions take place during the menstrual flow. Stress is also laid on the occurrence of the symptoms of pain, by which the amount of pain may be measured, *i.e.*, the occurrence of rapidity of the pulse, vomiting or severe shock, coldness and clamminess of the skin, &c. The absence of these would tend to the opinion that excessive pain was absent, and to some extent discount the neurotic element which so greatly complicates this question.

He draws attention to the spasmodic variety of menstrual pain and its frequent aggravation after marriage if pregnancy does not result, and attributes the pain to contraction of the internal os, associated with vasomotor spasm. With reference to treatment he suggests, as general measures suitable for most cases, regulation of the bowels and open-air exercise, notably horse riding and bicycling. For the spasmodic variety, heat to the extremities and surface, nitroglycerine, in doses of one-hundredth of a grain, and other diffusible stimulants, with ergot, antipyrin, bromides, and sal volatile; while, if medicinal treatment fails, thorough dilatation of the internal os, repeated at intervals if necessary.

In the congestive form local affections should be dealt with, but, unless the seat of actual disease, the ovaries should not be removed, as has been recommended and practised by some, since this has often no effect on the intermenstrual pain which is apt to accompany the affection. He does not consider that the mechanical theory of production in spasmodic dysmenorrhœa has any foundation in fact.

Lapthorn Smith,[38] on the other hand, attributes the frequency of occurrence of dysmenorrhœa in unmarried girls to the frequency of stenosis of the os uteri, which also is found in the unmarried. His treatment of dysmenorrhœa due to stenosis, after using measures to improve the general health, is by using acetanilide in ten grain doses three times a day, combined with stimulants, such as caffein citrate or whisky, to obviate too much general depression; but in the next sentence he says: "Opium and alcohol must be avoided." This apparently paradoxical statement probably refers to the administration of alcohol to relieve the pain, and not to its exhibition to counteract the depression of the acetanilide.

Failing these, he performs negative electrolysis, on which he has great reliance; or, as an alternative, dilates the cervix under anæsthesia, and curettes the uterine cavity, then applying iodised phenol, he lays great emphasis on the need for asepsis, and also on the possibility of accident, *e.g.*, rupture or perforation of uterus, in using this method. He disapproves entirely of the intra-uterine stem pessary. He states his results as 50 per cent. cured by dilatation, 12½ per cent. cured by electrolysis, and 5 per cent. cured by removal of appendages.

Gordon[39] advocates the method of negative electrolysis. He uses 60 cells of a Law battery and an electrode like an ordinary uterine sound, which is inserted into the cervical canal as far as it will go without pressure, and the current turned on until 15 or 20 milliampères are reached. Five or six minutes is long enough for each application.

Gardner and Lockhart[40] state that marriage diminishes the pain in the neuralgic variety, which is relieved or cured by pregnancy, and is not necessarily due to an accompanying ante-flexion, and draw attention to the fact that all pelvic symptoms are absent between the periods. An examination does not usually reveal any abnormality, and in the unmarried is uncalled for. In the congestive variety the pain is due to chronic or acute inflammatory mischief, and is associated with intermenstrual symptoms; here examination is imperative. In the obstructive variety the paroxysmal character of the pain is valuable for diagnostic purposes. Pelvic symptoms between the periods are at first absent, but appear later, and are usually due to secondary inflammatory processes. The neuralgic variety should be dealt with by general tonic treatment and open-air exercise, such as bicycling, and galvanism used if maldevelopment is present. The administration of morphia and alcohol is approved of.

Maddox[41] has found apioline, the active principle of petroselinum sativum, of great value in neurotic dysmenorrhœa. He insists that neuralgic cases may by neglect develop into cases of congestive or inflammatory dysmenorrhœa. The drug is administered in capsules, of which three a day should be taken, the remedy being used three days before the expected period. The amount of the drug contained in each capsule is not stated.

Mendes de Leon[42] combats the obstructive theory, and divides dysmenorrhœa into two classes—(1) Dysmenorrhœal endometritis, and (2) uterine spasm. The first class would include all forms in which any local mechanical obstacle could be made out to which the inflammatory changes in the endometrium are secondary. All other cases would fall under the head of uterine spasm. He regards the spasm as affecting the sphincter of the uterus, *i.e.*, the internal os.

Out of 37 cases of dysmenorrhœa under his care a local cause was found in 32; in the other five, certainly virgins, the affection was spasmodic. Twenty-one cases of manifest stenosis were observed, who had no pain or spasm; of 17 cases treated by curetting only one had stenosis to a high degree, eight were cured, seven were relieved but relapsed after a year, and two were not benefited; of the last, one was subjected to oöphorectomy and subacute oöphoritis was found. High amputation of the cervix by Schröeder's method was said to have cured one case of dysmenorrhœa and sterility.

The divergence of opinion among experts is strikingly illustrated by the previous quotations. Undoubtedly in cases of spasm dilatation of the cervix and curetting, if endometritis is present, offers the best chance of success. Electricity is no doubt valuable, but how far its value depends on mental influence appears uncertain; its application in the office of the physician and the performance of a subsequent journey, on foot or by train, by the patient, would soon lead to its abandonment, as cases of peritonitis have occurred after its use.

[37] Clinical Journal, March 3, 1897. [38] Amer. Med. Surg. Bulletin, April 24, 1897. [39] Amer. Med. Surg. Bulletin, April 10, 1897. [40] Amer. Med. Surg. Bulletin, July 10, 1897. [41] Charlotte Medical Journal, Aug., 1897. [42] Centralblatt für Gynäkologie, July 17, 1897.

